# Functional Benefits of Inpatient Cardiac Rehabilitation After Open Aortic and Valvular Surgery: A Retrospective Cohort Study

**DOI:** 10.3390/healthcare13151816

**Published:** 2025-07-25

**Authors:** Younji Kim, Suk-Won Song, Ha Lee, Myeong Su Kim, Seoyon Yang, You Gyoung Yi

**Affiliations:** 1 Department of Rehabilitation Medicine, Ewha Womans University Seoul Hospital, Ewha Womans University School of Medicine, 25, Magokdong-ro 2-gil, Gangseo-gu, Seoul 07804, Republic of Korea; yunji0114@naver.com (Y.K.); seoyonyang@gmail.com (S.Y.); 2 Thoracic and Cardiovascular Surgery, Ewha Womans University Aorta and Vascular Hospital, Ewha Womans University Medical Center, Seoul 07985, Republic of Korea; stevensong@ewha.ac.kr (S.-W.S.); manofwill@naver.com (H.L.); mmotion11@ewha.ac.kr (M.S.K.)

**Keywords:** inpatient recovery, physical performance, postoperative rehabilitation, aortic surgery, cardiovascular reconditioning

## Abstract

**Background/Objectives:** Patients undergoing open aortic and valvular surgery often experience postoperative deconditioning, yet research on the role of inpatient cardiac rehabilitation (CR) in this population remains limited. This study aimed to examine the effects of inpatient CR on muscle strength, mobility, psychological well-being, and quality of life in patients recovering from open aortic surgery. **Methods:** We conducted a retrospective study using the medical records of patients who participated in inpatient CR after open aortic surgery. Functional and psychological outcomes were evaluated using the Medical Research Council (MRC) sum score, Timed Up and Go (TUG) test, Five Times Sit-to-Stand test (5STS), Six-Minute Walk Distance (6MWD), Berg Balance Scale (BBS), Modified Barthel Index (MBI), Patient Health Questionnaire-9 (PHQ-9), and the EuroQol-5D (EQ-5D). Pre- and post-rehabilitation scores were compared to assess changes in functional status, mobility, and quality of life. A post-discharge satisfaction survey was also analyzed. **Results:** A total of 33 patients were included. Significant improvements were observed in MBI (*p* < 0.001), MRC sum score (*p* < 0.001), 6MWD (*p* < 0.001), BBS (*p* < 0.001), TUG (*p* = 0.003), 5STS (*p* < 0.001), EQ-5D (*p* = 0.011), and PHQ-9 (*p* = 0.009) following inpatient CR. Patients with lower baseline mobility (6MWD ≤ 120 m) exhibited greater improvement in MBI (*p* = 0.034). Of the 33 patients, 26 completed the satisfaction survey; most reported high satisfaction, perceived health improvements, and willingness to recommend the program. **Conclusions:** Inpatient CR following open aortic and valvular surgery resulted in significant gains in muscle strength, mobility, psychological health, and overall quality of life. Patients with greater initial impairment demonstrated especially notable functional improvement, supporting the value of tailored CR in this population.

## 1. Introduction

Patients undergoing aortic surgery, including procedures for aortic dissection, aortic aneurysm, and root replacement or aortic valve replacement, often experience significant postoperative deconditioning in physical capacity and mental health due to prolonged intensive care unit (ICU) stays and immobility [1,2,3]. Rehabilitation programs for patients after aortic surgery may facilitate functional recovery, and rehabilitation has been shown to enhance physical performance and play a critical role in maintaining long-term cardiovascular health [4,5,6,7,8]. Cardiac rehabilitation (CR) often consists of exercise training, lifestyle coaching, cardiovascular risk reduction, and psychosocial well-being [9].

Several studies have demonstrated the benefits of CR after cardiac and aortic surgeries. Sezai et al. [10] analyzed a large Japanese database and reported that inpatient CR led to shorter hospital stays, improved Barthel Index scores at discharge, and reduced medical costs in patients who underwent coronary artery bypass grafting (CABG), valve surgery, or aortic surgery. Although the benefits of CR have been extensively studied in patients with coronary artery disease, particularly those undergoing CABG, there is a relative paucity of evidence regarding the efficacy of rehabilitation in patients undergoing open aortic and valvular surgeries. These patients differ significantly in surgical complexity, postoperative recovery trajectory, and risk of complications. Open aortic and valvular surgeries are often more invasive, with longer operative times, higher ICU admission rates, and greater potential for musculoskeletal deconditioning.

Hirakawa et al. [1] and Zhou et al. [5] emphasized the importance of early mobilization in patients undergoing surgery for acute type A aortic dissection, showing that gradual CR under proper hemodynamic monitoring is both safe and effective. Shirai et al. [2] further highlighted that sitting at the bedside within two days postoperatively was associated with better independent ambulation outcomes. Despite growing clinical interest, there remains limited evidence evaluating the effect of inpatient CR on multidimensional outcomes such as muscle strength, balance, psychological well-being, and quality of life in this specific surgical population.

The present study aimed to evaluate whether structured inpatient CR following open aortic and valvular surgery leads to significant improvements in functional status, psychological well-being, and health-related quality of life. In addition, we sought to examine whether patients with lower baseline mobility experience greater functional gains from CR compared to those with relatively preserved mobility. To address these aims, we adopted a biopsychosocial framework of postoperative recovery and selected validated outcome measures representing three key domains. The primary outcome was the Modified Barthel Index (MBI), a widely used measure of independence in activities of daily living. Additional physical function measures included the Medical Research Council (MRC) sum score, Six-Minute Walk Distance (6MWD), and Five Times Sit-to-Stand test (5STS). Psychological status was assessed using the Patient Health Questionnaire-9 (PHQ-9), and quality of life was evaluated using the EuroQol 5-Dimension (EQ-5D). These instruments are commonly employed in cardiac and geriatric rehabilitation research and facilitate comprehensive evaluation of treatment effectiveness. We also assessed patient satisfaction with the CR program and collected qualitative feedback to guide potential service improvements.

By addressing this knowledge gap, our study aims to provide preliminary evidence supporting the implementation of individualized inpatient rehabilitation strategies after open aortic and valvular surgery. The findings may inform future care pathways and contribute to multidisciplinary models of postoperative recovery.

## 2. Materials and Methods

### 2.1. Study Population

**Figure 1:**
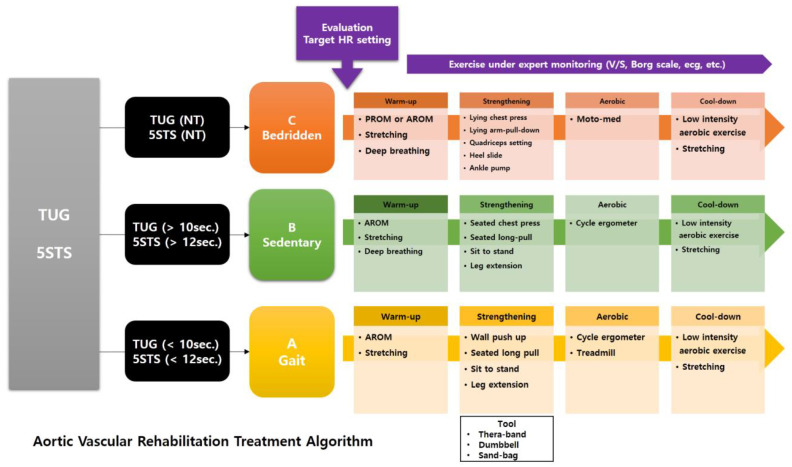
Inpatient cardiac rehabilitation programs after aortic surgery and functional stratification algorithm used to individualize cardiac rehabilitation programs.

In November 2024, the Ewha aortic and vascular hospital established the aortic vascular rehabilitation program, highlighting its critical role in accelerating post-surgical recovery (Figure 1). This retrospective study included patients who participated in the program between November 2024 and February 2025, when the standardized protocol had already been established and consistently applied. During this period, 40 patients were screened, and 33 patients were included in the final analysis. Seven patients were excluded due to incomplete records (*n* = 4) or inability to complete the program due to cognitive impairment or severe irritability (*n* = 3). Patients were identified from electronic medical records based on documented participation in the inpatient CR program following open surgery for aortic disease. As this was a retrospective pilot study based on the initial implementation of the rehabilitation program, no a priori sample size or power calculation was conducted. All consecutive eligible patients during the four-month period were included.

### 2.2. Ethics

This study was approved by the Institutional Review Board of Ewha Womans University Seoul Hospital (Approval Code: 2025-02-056; Approval Date: 5 March 2025). Although the inpatient CR program began in November 2024, no data were accessed or analyzed prior to IRB approval. Following approval, clinical and functional data were retrospectively collected from electronic medical records in accordance with the protocol. The study complied with the Declaration of Helsinki and institutional regulations for human subject research. As with all retrospective studies, this design may be subject to selection and information bias. To minimize such biases, we included all eligible patients during the predefined period and ensured consistent data extraction protocols.

### 2.3. Measures

We collected both objective and subjective data to evaluate patient characteristics, functional outcomes, and rehabilitation effects. Data were extracted from electronic medical records by two independent rehabilitation physicians and cross-verified by a third reviewer to ensure completeness and accuracy.

Objective outcomes included the MBI as the primary outcome, and secondary outcomes included the MRC sum score, 6MWD, Berg Balance Scale (BBS), Timed Up and Go (TUG) test, and 5STS test. The MBI has demonstrated high internal consistency with Cronbach’s alpha values around 0.90 [11]. Demographic and clinical variables—age, sex, comorbidities (e.g., hypertension, diabetes mellitus, chronic kidney disease), type of surgery, duration of intensive care unit (ICU) stay, use of mechanical ventilation, number of rehabilitation sessions, and duration of the rehabilitation program—were also extracted. Although comorbidities and rehabilitation duration were considered potential covariates, they did not show significant associations with baseline function or rehabilitation outcomes and were thus not further analyzed.

Subjective outcomes included the EQ-5D, PHQ-9, and a satisfaction questionnaire administered at discharge. The EQ-5D has shown acceptable reliability in clinical populations, with Cronbach’s alpha ranging from 0.73 to 0.85 [12]. The PHQ-9 is also a validated instrument with Cronbach’s alpha values between 0.86 and 0.89 [13]. Twenty-six patients completed the satisfaction survey, while seven did not due to early discharge or personal choice. All patients were included in the clinical outcome analysis regardless of survey completion. Due to the retrospective design of the study, blinding of outcome assessors was not feasible.

#### 2.3.1. Assessment of Muscle Strength and Physical Performance

Muscle strength and functional mobility were assessed before and after the rehabilitation programs. These included the MRC sum score for global muscle strength, Timed Up and TUG test, 5STS test, 6MWD, and gait speed. Patients were stratified into reduced and preserved mobility groups based on the median baseline 6 min walk distance (6MWD) of 120 m. This approach ensured a balanced comparison between groups and allowed for the analysis of rehabilitation effects relative to initial ambulatory capacity. Median-based stratification has been commonly applied in previous rehabilitation studies where standardized clinical cutoffs are unavailable [14,15]. For analytical purposes, patients were further stratified into reduced vs. preserved mobility groups based on the sample median of baseline 6MWD (120 m), as initial clinical groupings (A–C) did not show significant differences in outcome trajectories.

BBS scores were analyzed to assess balance and postural control before and after the rehabilitation programs.

#### 2.3.2. Assessment of Functional Independence, Quality of Life, and Patient Satisfaction

Functional independence was assessed using MBI scores. Quality of life was evaluated using the EQ-5D, and depressive symptoms were measured with the PHQ-9. Patient satisfaction with the rehabilitation program, perceived health improvement, and willingness to recommend the program to other patients were evaluated using a self-reported questionnaire. This questionnaire, which consisted of a 5-point Likert scale and an open-ended question to gather qualitative feedback on potential improvements, was administered after rehabilitation and before discharge. However, the questionnaire was not psychometrically validated, which may affect the reliability and generalizability of the subjective responses.

Because the study was retrospective, the questionnaire was administered only to patients who were available and willing to respond at the time of discharge. A total of 26 out of 33 patients completed the satisfaction survey, while 7 did not participate due to early discharge or personal choice. These individuals were still included in the clinical outcome analyses.

### 2.4. Cardiac Rehabilitation Protocol

To provide individualized CR treatment based on patients’ conditions, they were initially categorized into three groups (A, B, and C) according to their ability to walk or perform sit-to-stand tests.

Group A was defined as independent walkers—patients who could perform the TUG and 5STS tests without limitations. The rehabilitation program for this group primarily consisted of active range-of-motion (ROM) exercises, stretching, strengthening exercises, and aerobic exercises using both an active cycle ergometer and a treadmill.

Group B included patients who could walk with some limitations and were classified as assisted walkers. Their program comprised active ROM exercises, strengthening exercises, and aerobic exercise using an active cycle ergometer. Strengthening exercises were specifically designed so that Group A could perform them at a slightly higher intensity than Group B (Figure 1).

Patients who had difficulty with walking or performing sit-to-stand were assigned to Group C and defined as non-walkers. Their rehabilitation program included passive ROM exercises, stretching, deep breathing exercises, low-intensity strengthening exercises, and aerobic exercise using a passive cycle ergometer (MOTOmed^®,^ RECK-Technik GmbH & Co. KG, Betzenweiler, Germany).

The total number of inpatient rehabilitation sessions varied based on each patient’s clinical status and hospital discharge timeline. Daily rehabilitation was prescribed by a rehabilitation physician when patients were deemed medically stable. Thus, the number of sessions was not fixed but determined by individualized recovery trajectories. The average duration of the inpatient rehabilitation program was 13.5 ± 4.8 days, consisting of approximately 10.7 ± 4.9 sessions per patient. Each session lasted about 30 to 40 min. In most cases, rehabilitation was initiated within 5 days after surgery, depending on patient stability. All sessions were delivered by licensed physiotherapists and supervised by rehabilitation physicians to ensure safety and individualization.

By dividing the patients into these three groups, we developed individualized rehabilitation programs specifically tailored to each patient’s condition. All exercises were conducted under patient monitoring to ensure that the target heart rate (THR) was not exceeded, using the heart rate reserve (HRR) method.

### 2.5. Statistical Analysis

To assess the effectiveness of CR, statistical tests were selected based on the distribution of the data. The normality of continuous variables was evaluated using the Shapiro–Wilk test. For normally distributed variables, a paired *t*-test was used to compare pre- and post-rehabilitation values. For non-normally distributed variables, the Wilcoxon signed-rank test (Mann–Whitney U test for between-group comparisons) was applied. Based on the Shapiro–Wilk test, variables such as the MRC sum score and BBS demonstrated normal distributions; thus, we employed paired *t*-tests to assess pre- and post-CR improvements. Variables like the EQ-5D, which did not meet the normality assumption, were analyzed using the Mann–Whitney U test to evaluate changes before and after CR. Additionally, treatment effects between different functional groups were analyzed to identify variations in response to rehabilitation. Between-group comparisons were limited to unadjusted analyses, and we acknowledge the absence of multivariate control for potential confounders (e.g., age, comorbidities, rehabilitation days) as a limitation.

In addition to statistical significance, we calculated Cohen’s d to assess the effect size of pre–post differences. Cohen’s d quantifies the standardized mean difference, providing a measure of the practical significance of the observed changes. Effect sizes were interpreted as small (0.2), medium (0.5), or large (≥0.8), according to conventional thresholds. No correction for multiple comparisons (e.g., Bonferroni) was applied due to the exploratory nature of the study, which we acknowledge as a statistical limitation. Potential confounding factors (e.g., age, comorbidities, and surgery type) were reviewed and showed no significant differences between groups. Due to the small sample size and the pilot nature of this study, no multivariate analysis was performed. For all other analyses, a *p*-value ≤ 0.05 was deemed statistically significant. All statistical analyses were performed using SPSS (Version 29.0; IBM Corp., Armonk, NY, USA).

## 3. Results

### 3.1. Demographic Characteristics of Patients

The study included 33 patients who underwent inpatient CR after open aortic surgery or aortic valve replacement (AVR). As presented in Table 1, the mean age was 61.24 years (SD = 15.63), and the mean weight was 69.60 kg (SD = 15.92). Patients attended an average of 10.70 rehabilitation sessions (SD = 4.93). Baseline hemodynamic measurements showed a mean SBP of 131.09 mmHg (SD = 13.21), a mean DBP of 75.33 mmHg (SD = 11.54), and a mean resting HR of 82.89 (SD = 14.23). The types of surgeries performed in the 33 patients were as follows: replacement of the ascending aorta in 14 patients, replacement of the descending thoracic aorta (DTA) in 7 patients, AVR in 5 patients, replacement of the abdominal aorta in 5 patients, and other procedures in 2 patients.

### 3.2. Functional Status Changes After Cardiac Rehabilitation

As presented in Table 2, significant improvements were observed across all functional assessments. The MBI increased from 74.44 to a post-rehabilitation mean of 92.19, with a *p*-value of less than 0.001, indicating enhanced performance in activities of daily living. An MBI change of >10 points has been associated with clinically meaningful improvement [11], and our observed mean change of 17.75 points exceeds this threshold. The MRC sum score improved significantly from 35.97 to 47.21 post-rehabilitation (*p* < 0.001), reflecting increased muscle strength.

Psychological well-being, measured via the PHQ-9, demonstrated a reduction in mean scores from 7.88 to 3.45 after rehabilitation, with a *p*-value of 0.003, suggesting a decrease in depressive symptoms. The TUG times decreased from 19.25 s to 10.14 s (*p* = 0.003), and the 5STS times showed a reduction from 19.65 s to 10.14 s (*p* < 0.001).

Endurance and walking capacity, measured by the 6MWD, increased markedly from 142.88 m to 276.55 m post-rehabilitation. This 133.67 m improvement exceeds the minimal clinically important difference (MCID) of 54 m for cardiac patients [16]. Additionally, gait speed improved from 0.40 ± 0.37 m/s to 0.76 ± 0.39 m/s. Balance, as measured by the BBS, improved from 35.35 to 49.43 (*p* < 0.001). Quality of life, assessed using the EQ-5D, showed an increase from 0.70 to 0.86 (*p* = 0.001).

To evaluate the magnitude of observed changes, we calculated Cohen’s d effect sizes for all pre–post comparisons (Table 2). The results demonstrated large effect sizes across key functional domains, including MBI (d = 1.25), MRC sum score (d = 1.85), BBS (d = 0.95), and 6MWD (d = 0.98), indicating substantial clinical relevance. Additionally, psychological and quality of life measures such as PHQ-9 (d = −0.96) and EQ-5D (d = 0.93) also showed large effect sizes, supporting the effectiveness of inpatient CR.

### 3.3. Comparison of Functional Improvements Between Reduced and Preserved Mobility Groups

Patients were categorized into two groups based on their initial 6MWD: those who walked more than 120 m (the median value) were assigned to the preserved mobility group, while those who walked 120 m or less were assigned to the reduced mobility group. We compared improvements in the MRC sum score, BBS, 5STS, TUG test, 6MWD, gait speed, MBI, PHQ-9, and EQ5D between the two groups. Among the 33 patients, data for the 6MWD were missing for two individuals, and thus 31 patients were included in the analysis of this measure. Table 3 presents the comparison of functional improvements between the reduced mobility group (*n* = 17) and the preserved mobility group (*n* = 14) following inpatient CR.

The MBI improvement was significantly greater in the reduced mobility group compared to the preserved mobility group (12.10 ± 10.67 vs. 10.67 ± 7.07; *p* = 0.003). The BBS improvement was greater in the reduced mobility group (19.56 ± 12.50) than in the preserved mobility group (11.69 ± 9.05), showing a trend toward significance (*p* = 0.068). The 5STS improvement was significantly greater in the reduced mobility group (−15.07 ± 15.87 s) compared to the preserved mobility group (−3.42 ± 5.60 s, *p* = 0.035). TUG, 6MWD, gait speed, PHQ-9, and EQ5D showed improvements in both groups without significant differences.

### 3.4. Patient Satisfaction with Inpatient Cardiac Rehabilitation

Among the 26 patients who completed the post-rehabilitation satisfaction survey, 15 patients (58%) reported being “strongly satisfied”, 9 (35%) were “satisfied”, and 2 (8%) expressed a “neutral” level of satisfaction with the inpatient CR program. When asked about perceived health improvements after rehabilitation, 10 patients (38%) indicated a “strong improvement”, 13 patients (50%) reported a general “improvement in health”, and 3 patients (12%) responded neutrally. Regarding program recommendation, 11 patients (42%) stated they would “strongly recommend” the CR program, 14 (54%) said they would “recommend” it, and 1 patient (4%) remained neutral.

Patients provided several suggestions for enhancing the CR program. Some patients expressed a desire for a larger rehabilitation facility to accommodate more patients and improve accessibility. While many patients were satisfied with the program, some suggested incorporating more engaging and diverse educational lectures and activities to sustain interest and motivation. Acknowledging that dramatic functional improvement may not be achievable in a short period, patients valued structured movement opportunities over prolonged bed rest. Maintaining adherence to the structured rehabilitation plan was also emphasized as an important factor for optimizing patient outcomes.

## 4. Discussion

In the present study, we explored associations between inpatient CR on functional, psychological, and mobility outcomes in patients undergoing postoperative rehabilitation. Measures of physical function, including the MBI, MRC sum score and 6MWD, psychological well-being, and quality of life showed improvements post-rehabilitation. These findings align with previous studies highlighting the benefits of structured exercise programs in improving mobility, strength, and endurance in post-cardiac surgery patients [17,18,19]. In clinical practice, tailoring CR intensity and content based on pre-rehabilitation functional status can enhance patient engagement and outcomes. For instance, patients in the reduced mobility group demonstrated larger gains in MBI and 5STS, suggesting that early intervention targeting basic mobility and lower limb strength should be emphasized in this subgroup [14,15].

The increase in MRC sum scores was associated with improvement in muscle strength, which serves as the foundation for functional recovery [20]. Previous studies have highlighted the importance of resistance training in CR [21], as enhanced muscle strength is a critical component of mobility and endurance [5]. This improvement in strength was accompanied by significant gains in walking ability, as supported by increased 6MWD and improved gait speed. The 6MWD, which showed a statistically significant enhancement post-rehabilitation, is widely considered a key indicator of cardiovascular endurance, and its improvement has been associated with better long-term outcomes and reduced re-hospitalization rates [1]. These findings support the role of structured rehabilitation programs in supporting walking endurance and functional mobility [6]. This trend is consistent with previous findings in stroke and pulmonary rehabilitation cohorts, where those with more severe impairments at baseline had higher recovery margins [14,15,22]. These findings emphasize the importance of early mobility screening and functional stratification to optimize resource allocation and personalize CR intensity.

In alignment with the gains in strength and mobility, we observed significant improvements in postural control and balance, as reflected in the increasing BBS scores. Improving balance is particularly important for patients who underwent cardiac surgery [23], as impaired postural stability increases the risk of falls and further functional decline [24]. The improvements in BBS scores, along with decreased TUG times, suggest that CR may help alleviate post-surgical deconditioning and improve overall physical stability [4]. Prior research has demonstrated that patients who engage in rehabilitation programs incorporating balance training have shown reduced fall risk and enhanced mobility [25], both critical for long-term functional independence [23]. Notably, the enhancements in muscle strength, mobility, and balance cooperatively contributed to gains in functional independence, as reflected by the improvement in the MBI scores. Importantly, the magnitude of improvements observed in this study—particularly in MBI, 6MWD, and BBS—supports the clinical utility of initiating early CR even in patients with severe baseline impairments. For example, the observed mean increase of over 130 m in the 6MWD exceeds the established minimal clinically important difference (MCID) of 54 m in cardiac patients [16], indicating that the changes are not only statistically significant but also clinically meaningful. Similarly, the mean increase of nearly 18 points in MBI surpasses the commonly cited functional threshold of 10 points [11], underscoring its real-world impact on independence. Given that functional impairments are a major predictor of poor long-term outcomes in cardiac patients, our findings support the importance of structured CR in postoperative care protocols.

We observed a significant reduction in depressive symptoms following inpatient CR, with PHQ-9 scores decreasing from 7.88 to 3.45. Psychological distress, including depression and anxiety, is prevalent in patients recovering from major cardiac surgeries [5]. Our findings align with previous research highlighting the psychological benefits of CR, particularly in patients recovering from acute coronary events [26]. While prior studies have focused on ischemic heart disease, our results extend these findings to patients undergoing open aortic and valvular surgery, suggesting that CR may offer similar psychological benefits across different cardiac and vascular conditions. The observed reduction in depressive symptoms is likely caused by multiple factors, including structured physical exercise, psychological support, and social interaction, all of which are known contributing factors to mental health in cardiac patients [27].

In Korea, inpatient rehabilitation after cardiac surgery is not uniformly implemented and often varies by institution. Our findings suggest that structured inpatient CR could improve early functional recovery and patient satisfaction, indicating a need for systematic integration into postoperative care. Internationally, inpatient CR is already embedded in many healthcare systems, and introducing similarly structured pathways in Korea could reduce variability in care and improve outcomes. Policymakers should consider incorporating CR programs into standard postoperative care, with appropriate funding and guidelines to support sustainable implementation.

Specific recommendations for clinical integration include early postoperative initiation of CR (ideally within the first week of stabilization), prioritizing patients with reduced baseline mobility or psychological distress, and including core components such as individualized exercise, education, balance training, and psychological support. These elements should be standardized and adapted to the Korean healthcare setting. The development of national clinical guidelines is warranted to define clear indications, timing, and content of CR programs after aortic or valvular surgery.

### 4.1. Comparison of Functional Improvements Between Reduced and Preserved Mobility Groups

In the present study, we categorized patients into two groups based on their initial 6MWD before CR and analyzed their functional improvements following inpatient CR. The findings suggest that while both groups experienced significant improvements, those with lower baseline mobility (reduced mobility group) demonstrated greater enhancement in MBI and 5STS measures. In clinical practice, tailoring CR intensity and content based on pre-rehabilitation functional status can enhance patient engagement and outcomes. For instance, patients in the reduced mobility group demonstrated larger gains in MBI and 5STS, suggesting that early intervention targeting basic mobility and lower limb strength should be emphasized in this subgroup. The significantly greater improvement in MBI within the reduced mobility group indicates that patients with poorer baseline functional status obtained more benefits from CR [21]. This aligns with previous findings suggesting that patients with greater impairments at baseline show more pronounced functional recovery following structured CR [17]. Given that MBI is a strong predictor of functional independence, these results highlight the importance of individualized CR strategies to maximize outcomes in functionally compromised patients [28].

The improvement in BBS scores was greater in the reduced mobility group (19.56 ± 12.50) than in the preserved mobility group (11.69 ± 9.05); this difference approached statistical significance (*p* = 0.068). This suggests that individuals with lower baseline mobility may experience more enhancement in dynamic balance and postural control following structured CR. Additionally, 5STS improvement, which reflects lower limb strength and functional endurance, was significantly greater in the reduced mobility group (−15.07 ± 15.87 s) compared to the preserved mobility group (−3.42 ± 5.60 s, *p* = 0.035). This finding underscores the potential for inpatient intensive rehabilitation to yield substantial benefits in lower limb function, particularly for patients with greater initial impairments. These results are consistent with previous studies suggesting that patients with lower functional status at baseline tend to achieve relatively greater improvements following rehabilitation, likely due to their higher capacity for recovery and neuromuscular adaptation [29,30].

Our subgroup analysis showed that patients with lower initial mobility experienced greater functional gains from inpatient CR. This is consistent with previous studies reporting that individuals with poor baseline function may benefit more from structured rehabilitation programs [31]. This phenomenon aligns with the “ceiling effect” in recovery, where those with lower starting function have greater potential for measurable improvement [32]. Incorporating this understanding into patient selection may help optimize CR delivery.

Our findings support the early implementation of structured inpatient CR programs after aortic and valvular surgery, particularly for patients with low initial mobility. Clinicians should assess patients’ functional status early postoperatively to identify those most likely to benefit from intensive CR. Moreover, simple patient-reported satisfaction metrics, even if not validated, may be useful to monitor program effectiveness and encourage engagement. Real-world adoption of CR should involve standardized protocols, interdisciplinary teams, and early initiation.

The integration of psychological support and balance training within CR programs may also be critical, particularly for those with higher depressive burden or impaired postural stability. These components should be standardized within CR protocols for post-aortic surgery patients. Future research is needed to investigate whether individualized CR protocols based on initial functional status could improve recovery trajectories and enhance long-term outcomes in open aortic surgery patients.

### 4.2. Patient Satisfaction with Inpatient Cardiac Rehabilitation

The survey results demonstrate a high level of satisfaction for the CR program in this population. The majority of patients expressed strong satisfaction with the program, perceived improvements in their health status, and reported a high likelihood of recommending the program to others. These findings align with previous studies reporting that structured CR programs contribute to increased patient satisfaction and perceived health benefits [21]. The positive feedback suggests that the program effectively addressed patients’ needs, possibly through individualized rehabilitation plans, structured exercise sessions, and continuous medical supervision [4,33]. Moreover, previous research has demonstrated that higher satisfaction with rehabilitation programs is associated with better adherence and improved functional outcomes [34]. Despite the overall positive results, a few patients reported moderate satisfaction, indicating potential room for improvement. Previous studies have emphasized the importance of psychological support, patient education, and personalized goal-setting in optimizing patient satisfaction and long-term engagement [34]. Future studies should investigate specific factors affecting patient satisfaction, such as accessibility, program duration, and individual patient expectations, to increase compliance and maximize benefits.

The suggestions provided from the patients offer valuable insights into potential improvements. Firstly, while overall satisfaction was high, some patients recommended the incorporation of more engaging and diverse educational lectures and activities. Enhancing the variety and interactivity of the educational components could maintain patient interest and motivation throughout the CR. This might include multimedia presentations, interactive workshops, or personalized educational modules that meet individual learning needs. This perspective is consistent with current evidence highlighting the importance of early mobilization and consistent physical activity for long-term functional improvement. Additionally, compliance with structured rehabilitation plans has emerged as an important factor in optimizing patient outcomes. The emphasis on adherence suggests that the success of the program depends not only on the initial design but also on the ongoing efforts of patients to follow the prescribed regimen. Strategies to enhance compliance such as regular follow-up sessions, motivational interviews, or digital reminders could be explored to improve outcomes. Our findings contribute to the growing body of evidence supporting individualized rehabilitation frameworks that incorporate mobility stratification, multimodal training, and psychosocial support.

### 4.3. Study Limitations

Despite the encouraging results, several limitations should be acknowledged. First, the relatively small sample size (*n* = 33) limits the generalizability of our findings and increases the potential for Type I and Type II errors. Although we employed appropriate statistical methods considering the data distribution, conclusions drawn from such a small cohort should be interpreted with caution. Given the exploratory and pilot nature of this study and the limited sample size, corrections for multiple comparisons (e.g., Bonferroni adjustment) were not applied. We acknowledge that this may increase the risk of Type I error (α inflation), and the results should be interpreted with caution. As a retrospective study without a comparator group, causal relationships between inpatient CR and the observed improvements cannot be firmly established. The observed associations may reflect selection bias or regression to the mean, rather than true treatment effects. Furthermore, potential confounding variables—such as type of surgery, comorbidities, and rehabilitation duration—were not controlled for, which may have influenced the outcomes. Multivariate analysis or matching techniques were not applied due to the limited sample size, which is a recognized limitation. The absence of long-term follow-up also limits our ability to assess the sustainability of these improvements after discharge.

The satisfaction survey used to evaluate subjective experiences was custom-developed for this study and was not based on a previously validated instrument. Therefore, the absence of formal psychometric testing may affect the reliability and interpretability of the reported satisfaction levels. In addition, the patient satisfaction survey used in this study was developed by the authors and has not been previously validated, which may affect the reliability of subjective data. Nevertheless, this study provides preliminary evidence supporting the feasibility and potential benefits of structured inpatient CR in patients undergoing open aortic and valvular surgery. Future randomized controlled trials with larger sample sizes and longer follow-up are warranted to confirm these findings and optimize rehabilitation protocols for this specific patient population.

## 5. Conclusions

In summary, the results suggest that structured inpatient CR is effective in improving physical function, mobility, and psychological well-being in patients recovering from open aortic and valvular surgery. Notably, patients with greater baseline impairments achieved more substantial gains, highlighting the value of early assessment and tailored interventions. These findings indicate that inpatient CR should be prioritized for patients with reduced mobility, deconditioning, or depressive symptoms after surgery. Early screening using simple functional tests such as 6MWD and 5STS may help clinicians identify those who would benefit most. From a healthcare system perspective, structured inpatient CR programs should be integrated into routine postoperative care to ensure equitable access and reduce variability in outcomes. Standardized protocols, interdisciplinary teams, and funding mechanisms are essential to achieve this goal. While promising, our findings are based on a small, retrospective cohort without a control group. Future prospective studies with larger samples and longer follow-up are warranted to validate these results and assess long-term effects on readmission and quality of life. The majority of patients expressed strong satisfaction with the CR and stated they would recommend the CR program to others. High levels of patient satisfaction further support the acceptability and perceived benefit of the program. These results emphasize the need to incorporate individualized inpatient CR into standard postoperative care pathways to enhance recovery and promote functional independence in this high-risk population. In conclusion, this study provides preliminary evidence supporting the integration of individualized inpatient CR into standard postoperative care pathways, particularly for high-risk patients undergoing open aortic or valvular surgery.

## Figures and Tables

**Table 1:** Baseline characteristics of patients who underwent inpatient cardiac rehabilitation.

Variable	Total (*n* = 33)
Age, yr	61.2 ± 15.6
Male/Female	24/9
Weight, kg	69.6 ± 15.9
Height, cm	163.3 ± 16.1
Systolic Blood Pressure, mmHg	131.1 ± 13.2
Diastolic Blood Pressure, mmHg	75.3 ± 11.5
Resting Heart Rate, bpm	82.9 ± 14.2
Number of Rehabilitation Sessions	10.7 ± 4.9
ICU Days	3.4 ± 2.1
Length of Hospital Stay, d	28.9 ± 12.4
Operation	*n*
Replacement of Ascending Aorta	14
Replacement DTA	7
Replacement of Aortic Valve	5
Replacement of Abdominal Aorta	5
Others	2

Abbreviations: ICU, intensive care unit; DTA, descending thoracic aorta. Footnote: Data are presented as mean ± SD or *n*.

**Table 2:** Functional status comparison before and after cardiac rehabilitation (*n* = 33).

Measure	Pre (Mean ± SD)	Post (Mean ± SD)	*p*-Value	Cohen’s d	Pre, Median (Min–Max)	Post, Median (Min–Max)
MBI	74.44 ± 17.77	92.19 ± 9.47	<0.001	1.25	79 (37–98)	98 (66–100)
MRC sum score	35.97 ± 2.34	47.21 ± 8.29	<0.001	1.85	36 (33–48)	48 (33–60)
PHQ-9	7.88 ± 6.03	3.45 ± 2.53	0.003	−0.96	6 (0–22)	4 (0–14)
TUG, s	19.25 ± 17.41	10.14 ± 6.26	0.003	−0.70	13.75 (6.70–92.00)	8.50 (6.00–32.30)
5STS, s	19.65 ± 14.05	10.14 ± 5.67	<0.001	−0.89	15.00 (7.00–78.00)	8.85 (5.50–35.00)
6MWD, m	142.88 ± 133.27	276.55 ± 140.28	<0.001	0.98	120.00 (0–451.00)	303.00 (38.00–485.00)
Gait speed, m/s	0.40 ± 0.37	0.76 ± 0.39	<0.001	0.95	0.33 (0–1.25)	0.84 (0.11–1.35)
BBS	35.35 ± 16.01	49.43 ± 13.67	<0.001	0.95	42.50 (0–54.00)	55.00 (3.00–56.00)
EQ-5D	0.70 ± 0.23	0.86± 0.08	<0.001	0.93	0.77 (0.15–0.95)	0.86 (0.68–0.95)

**Table 3:** Comparison of functional improvements between reduced and preserved mobility groups.

Measure	Reduced Mobility Group (*n* = 17)	Preserved Mobility Group (*n* = 14)	*p*-Value
MRC sum score improvement	10.65 ± 8.00	12.71 ± 7.43	0.233
BBS improvement	19.56 ± 12.50	11.69 ± 9.05	0.068
5STS improvement, s	−15.07 ± 15.87	−3.42 ± 5.60	0.035
TUG improvement, s	−3.41 ± 21.58	−9.53 ± 5.60	0.675
6MWD improvement, m	139.57 ± 54.60	120.58 ± 51.85	0.688
Gait speed improvement, m/s	0.40 ± 0.35	0.33 ± 0.14	0.233
MBI improvement	12.10 ± 10.67	10.67 ± 7.07	0.003
PHQ-9 improvement	1.45 ± 6.47	−1.18 ± 1.60	0.743
EQ5D improvement	0.14 ± 0.24	0.18 ± 0.16	0.473

Footnote: Data are presented as mean ± SD.

## Data Availability

Data are available from the corresponding author upon reasonable request.
